# Estimating the risk of gastrointestinal illness associated with drinking tap water in Norway: a prospective cohort study

**DOI:** 10.1186/s12889-024-19607-2

**Published:** 2024-08-05

**Authors:** Susanne Hyllestad, Trude Marie Lyngstad, Jonas Christoffer Lindstrøm, Richard Aubrey White, Monica Andreassen, Camilla Svendsen

**Affiliations:** 1 Department of Infection Control and Preparedness, Norwegian Institute of Public Health, P.O. Box 222, Skøyen, Oslo, 0213 Norway; 2 Department of Method Development and Analytics, Norwegian Institute of Public Health, Oslo, Norway; 3 Department of Infection Control and Vaccines, Norwegian Institute of Public Health, Oslo, Norway; 4 Department of Chemical Toxicology, Norwegian Institute of Public Health, Oslo, Norway

**Keywords:** Waterborne infections, Drinking water, Cohort study, Risk of gastrointestinal infection, Tap water consumed

## Abstract

**Background:**

The delivery of safe drinking water has high public health relevance, as reflected in the Sustainable Development Goals (SDG6). Several precautionary actions have reduced the burden associated with infectious diseases in high-income countries; however, pollution in source waters, inadequate disinfection, and premise plumbing, along with an increased awareness that intrusion in the drinking water distribution system, represents risk factors for gastrointestinal illness linked to consume of drinking water. Sporadic cases of waterborne infections are expected to be underreported since a sick person is less likely to seek healthcare for a self-limiting gastrointestinal infection. Hence, knowledge on the true burden of waterborne diseases is scarce. The primary aim with the present study was to estimate the risk of gastrointestinal illness associated with drinking tap water in Norway.

**Methods:**

We conducted a 12-month prospective cohort study where participants were recruited by telephone interview after invitation based on randomised selection. A start up e-survey were followed by 12 monthly SMS questionnaires to gather information on participants characteristics and drinking tap water (number of 0.2L glasses per day), incidence, duration and symptoms associated with gastrointestinal illness. Associations between the exposure of drinking tap water and the outcome of risk of acute gastrointestinal illness (AGI) were analysed with linear mixed effects models. Age, sex, education level and size of the drinking water supply were identified as potential confounders and included in the adjusted model.

**Results:**

In total, 9,946 persons participated in this cohort study, accounting for 11.5% of all invited participants. According to the data per person and month (99,446 monthly submissions), AGI was reported for 5,508 person-months (5.5 per 100 person-months). Severe AGI was reported in 819 person-months (0.8 per 100 person-months). Our study estimates that 2–4% of AGI in Norway is attributable to drinking tap water.

**Conclusions:**

This is the largest cohort study in Norway estimating the burden of self-reported gastrointestinal infections linked to the amount of tap water drunk in Norway. The data indicate that waterborne AGI is not currently a burden in Norway, but the findings need to be used with caution. The importance of continued efforts and investments in the maintenance of drinking water supplies in Norway to address the low burden of sporadic waterborne cases and to prevent future outbreaks needs to be emphasised.

**Supplementary Information:**

The online version contains supplementary material available at 10.1186/s12889-024-19607-2.

## Background

The delivery of safe drinking water has a high public health relevance, as reflected in the Sustainable Developments Goal (SDG) 6 for accessing safe water for all [1]. Several precautionary actions have reduced the burden associated with infectious diseases in high-income countries, particularly due to the expansion of basic services such as drinking water and sanitation [2]. However, waterborne infection may be caused by pollution in source waters, inadequate disinfection, and premise plumbing [3] and the risks of gastrointestinal illness linked to consume of contaminated drinking water is higher than they should be despite improving the level of safely managed drinking water supply [4]. In addition, there is increased awareness that the drinking water distribution system itself represents a risk factor for gastrointestinal illness [5] since loss of pressure in the distribution system may result in recontamination of pathogenic viruses, bacteria and parasites in drinking water [6]. A loss of pressure in the supply system can lead to pathogenic viruses, bacteria and parasites entering water sources, distribution systems or both in various ways and may cause outbreaks [5]. Ageing pipe infrastructure in particular is vulnerable to the backflow of contaminants during pressure loss [7]. It is a challenge to inspect the condition of the water distribution system and evaluate the risk of intrusion of contaminated water. Technological advances in terms of real-time monitoring of water distribution systems suggest the potential for an earlier warning of contamination events; however, deploying such measures may be challenging when linking monitoring data to operational response [8]. In addition, if a contamination event occurs, there is no necessary efficient water treatment (hygienic barriers) before drinking water reaches households.

Sporadic cases of waterborne infections are expected to be underreported since a sick person is less likely to seek healthcare for a self-limiting gastrointestinal infection [9]; therefore, notified cases represent only the ‘tip of the iceberg’ [10]. Hence, the true burden of waterborne diseases in high-income countries is not known. Several studies have been conducted to determine the disease burden attributed to drinking water in high-income countries [11, 12]. It may challenging to identify the source of infection for gastrointestinal illness on an individual level and in order to rule out causes of the disease other than drinking water, for example contaminated food or lack of hygiene. To overcome confounding factors, randomised controlled trials (RCTs) have been conducted in Canada [13], the United States (US) [14] and Australia [15], reporting different results on the association between tap water consumption and illness. A positive association was reported in Canada, whereas a correlation was not found in the US or Australia. A possible explanation for these differences may be related to the study design and contextual study area, thus highlighting the challenge in estimating the burden of waterborne diseases; although randomised controlled studies are regarded as the ‘gold standard’ for studying causal relationships, they do not necessarily provide results relevant for drinking water supplies in general [16].

In Norway, regulated drinking water supplies serve approximately 90% of the population and are generally considered to be of good quality, with high levels of compliance with water quality standards [17]. However, the risk of contamination in the distribution system has become a growing concern in Norway in recent years, along with an awareness that an aging pipe infrastructure is vulnerable to the backflow of contamination during the loss of pressure [7]. According to statistics reported from water supply systems, Norway has a leakage of approximately 33%, ranging from 20 to 80%, of the produced drinking water [18], which is significantly greater than that of other countries [19]. In Sweden, the level of leakage is estimated to be 20%; in Denmark, it is approximately 10%; and in the Netherlands, it is as low as 5% [20]. Yearly, planned and spontaneous breaches in the distribution system are reported in Norway, causing low-pressure situations where contaminated water may enter the water pipeline [21]. Such intrusion events have been associated with gastrointestinal illness [7, 22]. When anticipating the current pace of renewing drinking water pipelines, it is estimated that it will take approximately 145 years to upgrade the drinking water pipe network in Norway [18]. The effects of changing climatic factors are expected to act as stressors to aging and vulnerable drinking water supply systems with potential health consequences [23, 24]. It is anticipated that more frequent heavy rainfall and flood events will affect Norway [25]. Strong evidence points to an association between climatic factors, such as heavy rainfall, and food and waterborne diseases, such as salmonellosis and campylobacteriosis, in the sub-Arctic region [23]. Concern about the ability of small water supply systems to manage a water crisis for effective public health protection is also a concern due to a lack of financial, managerial and competent resources [26]. These factors underscore the importance of monitoring the burden of disease related to drinking water.

In terms of the disease burden of waterborne cases, studies reveal that waterborne outbreaks occur each year in Norway [27], and 4,000–8,000 cases related to food and waterborne pathogens are reported to the Norwegian Surveillance System for Communicable Diseases (MSIS) annually [28]. Large waterborne outbreaks are usually investigated [29–31], but underreporting of smaller outbreaks is assumed. Outbreaks affecting few people, which is the case for outbreaks where the source is contamination of small waterworks, private wells, or parts of the distribution system, are probably not reported or investigated and are therefore underreported. Gastrointestinal illnesses diagnosed by primary health care are registered in the Norwegian Syndromic Surveillance System (NorSySS), although it is not possible to distinguish waterborne disease from diseases caused by other sources such as food. Research revealed an association between heavy precipitation events and waterborne outbreaks in Nordic countries for single households, with groundwater serving as the raw water source during summer [32]. Two population-based studies have investigated the burden of gastrointestinal illness in Norway [33, 34]; however, both studies provide uncertain estimates and are outdated.

With the backdrop of underreporting of sporadic waterborne cases nationally, the overall aim of this study was to assess the association between the amount of tap water drunk (glasses per day) and the risk of gastrointestinal illness in Norway and to estimate the disease burden of waterborne infections.

## Materials and Methods

### Study context

The study was conducted between 2018 and 2020. Norway is a relatively small country in the Nordic region, with approximately 5.4 million registered inhabitants as of November 2023 [35]. The population is distributed throughout the country and is divided into approximately five of the largest urban and rural settlements [36]. Norway is a high-income country that has the highest living standard in the Organisation for Economic Co-operation and Development (OECD) area [37]. In Norway, there are approximately 1,500 drinking water supply systems serving households that are geographically widespread. Many of these areas are managed by small drinking water organisations. Approximately 86% of the water supplies serve fewer than 5,000 residents, while a few large drinking water supplies serve most residents living in the largest cities and urban areas. Since the middle of the 1990s, several hygienic barriers have been implemented to ensure safe drinking water in a targeted programme to improve the quality of drinking water in Norway [38]. Today, only a small proportion of consumers of public drinking water receive water that is not disinfected [38]. A typical drinking water supply system in Norway makes use of surface water as a raw water source, serving 90% of the connected population, and as few as 10% are served by water supplies using ground water as a raw water source. Safe drinking water from surface water is ensured by establishing a deep and protected intake in the lake and filtration and coagulation to remove particles associated with parasitic protozoa, UV radiation and adjustment of pH for corrosion control in pipelines [38].

### Study design, case definition and study population

The study was undertaken as a 12-month prospective cohort study, drawing inspiration from a study in the municipality of Ale in Sweden [39]. Acute gastrointestinal infection (AGI) was defined as a case in which the respondent reported at least one of the following: (i) three or more occurrences of diarrhea or (ii) vomiting within 24 h. Severe AGI vas was defined as five or more occurrences of diarrhea within 24 hours. The study population included persons aged 0–80 years who were living in Norway and served by selected drinking water supplies (see below). In total, 5,128,362 persons aged 0–80 years were residents in 2019, according to Statistics Norway [35].

### Selection of drinking water supplies, invitation, and recruitment of participants

Drinking water supplies were the basis for recruiting cohort participants (Table 1). All Norwegian drinking water supplies serving 50–999 persons, a randomised selection of drinking water supplies serving 1,000–5,000, 5,001–19,999 or 20,000–100,000 persons, and all drinking water supplies serving more than 100,000 persons were invited to participate in the study and to provide post addresses of their respective households. One person, aged 0–80 years, was thereafter selected randomly per household. In total, 86,226 participants were selected and received a postal invitation.

**Table 1:** Selected drinking water supplies, invited, and recruited participants

Water supply category	Small water supplies	Large water supplies				Total
Persons supplied	50–999	1,000–4,999	5,000–19,999	20,000–100,000	> 100,000	
Norwegian water supplies	1,185	250	110	51	5	1,601
Selected water supplies (%)	374 (32%)	31 (12%)	10 (9%)	5 (10%)	5 (100%)	425 (27%)
Invited participants^a^	26,559	14,417	17,615	13,817	13,818	86,226
Recruited participants (%)	2,352 (8.9%)	1,388 (9.6%)	1,977 (11.2%)	2,023 (14.6%)	2,214 (16.0%)	9,954 (11.5%)

^a^Participants receiving postal invitation

Invited participants were recruited through telephone interviews conducted by Kantar TNS [40] from December 2018 to February 2020. If the invited participant was younger than 16 years, one of the parents was contacted and interviewed on behalf of the child/adolescent. Individuals suffering from chronic gastrointestinal illness and/or who were week commuters were excluded from participation.

### Data collection

The protocols for telephone interviews, electronic surveys (e-surveys), and text message surveys (SMS questionnaires) were developed by the Norwegian Institute of Public Health. An e-survey was employed to gather information on gastrointestinal illness and factors that may influence water consumption and consequently the frequency of AGI. The e-survey included information per participant on sex, age, education and residence. The e-survey was followed by SMS questionnaires to collect monthly data for 12 months per participant on the amount of tap water drunk (number of 0.2L glasses) the last 24 h, incidence, duration and symptoms associated with gastrointestinal illness, and date of submission. Translated versions of interviews and SMS questionnaires to English are presented in Supplementary material.

Information about the study was advertised by general information campaigns and by mailed information brochures to the individuals who agreed to participate in the study before the data collection (interviews/e-surveys/SMS questionnaires). The selection of participants, recruitment, and data collection (e-survey, SMS questionnaires) were carried out by subcontractors: Evry [41] (identifies participants’ address and contact information) and Kantar TNS (carried out telephone interviews and SMS questionnaires). Information about the study was advertised by general information campaigns and by mailed information brochures to the individuals who agreed to participate in the study before the data collection (interviews/e-surveys/SMS questionnaires). Data on drinking water supplies (drinking water organisation ID and size) were retrieved from the Norwegian Registry of Drinking Water Supplies and linked to the interview data [42].

### Statistical analysis

Associations between the amount of tap water drunk (exposure) and monthly AGI or severe AGI per person (outcome) were analysed with linear mixed effects models. A random intercept was included for each subject. Tap water drunk was included as a fixed effect. A linear regression model was chosen to allow for the estimation of risk difference (RD). Models were run with tap water drunk both as categorical and continuous variables. Potential confounders such as age, sex, education level and size of the drinking water supply were identified by directed acyclic graph (DAG), *i.e.,* variables related to both exposure and outcome; thus, these variables were included in the adjusted model. In addition, we included the month of the response to account for potential seasonal effects. AGI attributable to drunk tap water was estimated by combining the effect estimates from the regression models with 1) exposure data from the survey, and 2) a counterfactual population where no one drinks tap water. These two estimates were then subtracted from each other.

R version 4.3.0 (The R Foundation for Statistical Computing) was used to analyse the data, and the lme4 package was used for fitting mixed effects models [43].

The STROBE reporting guidelines for observational studies [44] and the Declaration of Helsinki (2013) were followed in the design and reporting of this study.

## Results

### Response

A total of 86,226 persons were invited to participate in the study, and 9,954 (11.5%) responded and completed the start-up questionnaire (e-survey) (Table 1). Over the study period, the participants answered 103,683 monthly questionnaires. A total of 4,237 (4.1%; 126 participants) monthly questionnaires were excluded because 1) the respondents had reported consuming an unrealistic amount (≥ 30 glasses/6 L) of tap water in the last 24 h (58 questionnaires), and/or 2) the participant did not report tap water consumption (4,179 questionnaires). Ultimately, 507 of the 9,954 participants who completed the start-up questionnaire did not complete any monthly questionnaires and were therefore excluded. This left us with 9,447 participants who answered at least one monthly questionnaire, for a total of 99,446 monthly questionnaires.

**Fig. 1:**
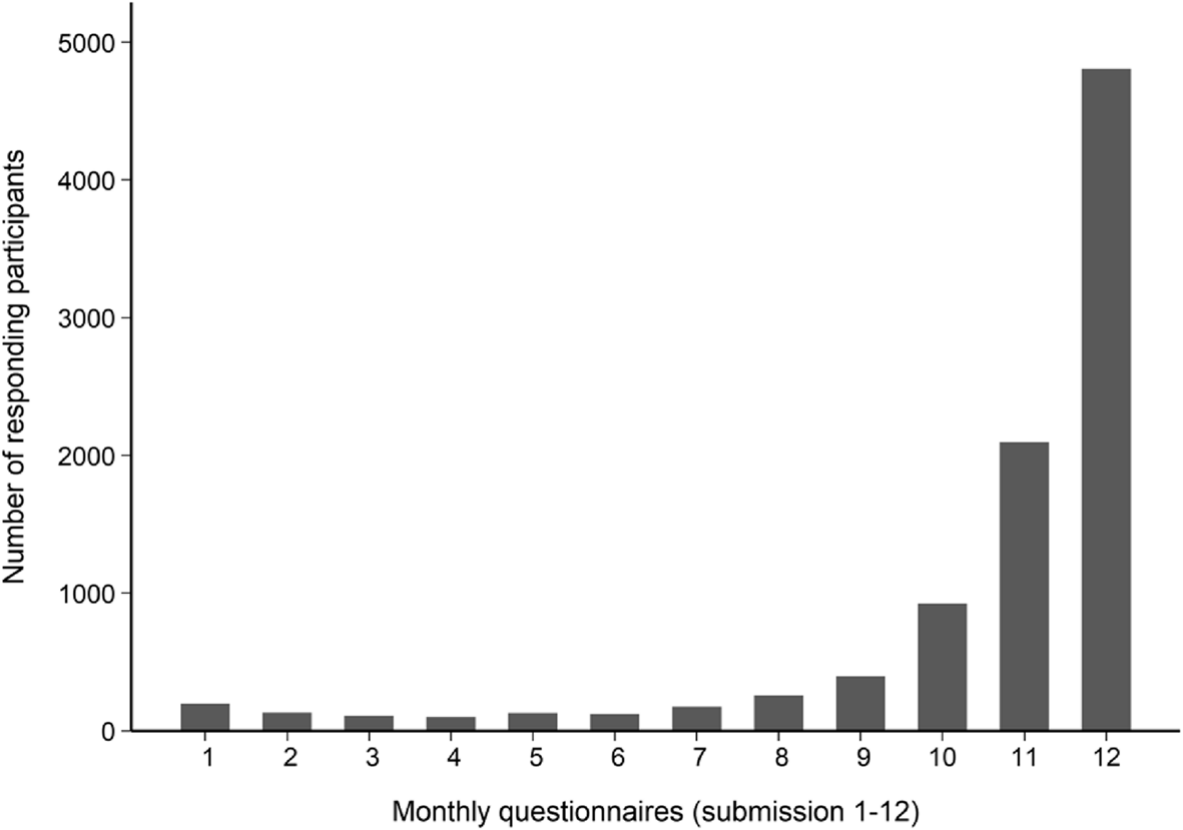
Number of responding participants per monthly questionnaire (submission 1–12)

Among the 9,447 participants, 83% (7,832 participants) submitted monthly questionnaires for at least 10 months, and 51% (4,809 participants) submitted all 12 months (Fig. 1).

### Cohort characteristics

Among the 9,447 participants, 89% (8,383 participants) were 19 years or older, and 53% (4,993 participants) were female. Seventy-six percent (7,223 participants) received water from large drinking water supplies, and 24% (2,224 participants) received water from small drinking water supplies. Geographical distribution (definition in Table 2): Regions East and West were the regions with the highest proportion of participants among those invited; 61% (5,774 participants) and 22% (2,046 participants) lived in Regions East and West, respectively. Fifty-one percent (4,827 participants) reported having tertiary education, and 38% (3,585 participants) reported having primary, secondary, or other education. Eleven percent (1,004 participants) were under 18 years of age and presumably still in primary or secondary education (Table 2).

**Table 2:** Characteristics of the participants (one per household, 0–80 years) in the Norwegian longitudinal cohort study divided by small (50–1,000 persons supplied) and large (> = 1,000 persons supplied) water supplies

	Small water supplies	Large water supplies	Total	National comparison
**Total**	2,224	7,223	9,447	5,128,362
**Tap water drunk**^a^				
0–1 glasses	90 (4%)	300 (4%)	390 (4%)	-
2–3 glasses	561 (25%)	1818 (25%)	2379 (25%)	-
4–5 glasses	869(39%)	2703 (37%)	3572 (28%)	-
6–7 glasses	446 (20%)	1486 (21%)	1932 (20%)	-
8 + glasses	258 (12%)	916 (13%)	1174 (12%)	-
**Sex**				
Male	1,068 (48%)	3,386 (47%)	4,454 (47%)	50%
Female	1,156 (52%)	3,837 (53%)	4,993 (53%)	50%
**Age**				
0–5	52 (2%)	295 (4%)	347 (4%)	7%
6–12	79 (4%)	390 (5%)	469 (5%)	9%
13–18	56 (3%)	192 (3%)	248 (3%)	7%
19–49	588 (26%)	2,156 (30%)	2,744 (29%)	43%
50–64	877 (39%)	2,397 (33%)	3,274 (35%)	19%
65–80	572 (26%)	1,793 (25%)	2,365 (25%)	14%
**Education**				
Tertiary education	892 (40%)	3,935 (54%)	4,827 (51%)	26%
Primary, secondary, other education	1,152 (52%)	2,433 (34%)	3,585 (38%)	52%
Persons below 18 years	171 (8%)	833 (12%)	1,004 (11%)	22%
Missing	9 (0,4%)	22 (0,3%)	31 (0,3%)	-
**Region**^b^				
South	151 (7%)	0 (0%)	151 (2%)	6%
East	452 (20%)	5,322 (74%)	5,774 (61%)	51%
West	876 (39%)	1,170 (16%)	2,046 (22%)	26%
Middle	232 (10%)	546 (8%)	778 (8%)	9%
North	513 (23%)	185 (3%)	698 (7%)	9%

^a^Mean number of 0.2L glasses (per 24h) reported per month and participant

^b^South: County of Aust-Agder and Vest-Agder; East: County of Østfold, Akershus, Oslo, Hedmark, Oppland, Buskerud, Vestfold and Telemark; West: County of Rogaland, Hordaland, Sogn-og-fjordane and Møre and Romsdal; Middle: County of Trøndelag; North: County of Nordland, Troms and Finnmark

### Acute gastrointestinal infection (AGI), severe AGI and water consumption

According to the data per person and month (99,446 monthly submissions), AGI was reported for 5,508 person-months (5.5 per 100 person-months). Severe AGI was reported in 819 person-months (0.8 per 100 person-months). The reported number of person-months with AGI or severe AGI varied somewhat by sex, age, education level and calendar month. The highest number of reported AGIs was found in individuals aged 0–5 years (342 person-months; 9.5 per 100 person-months), followed by individuals aged 19–49 years (2,066 person-months; 7.6 per 100 person-months) (Table 3). Overall, the mean number of glasses of water drunk per day per person was 4.9 (median = 4).

**Table 3:** Reported number of months with acute gastrointestinal infection (AGI) and severe AGI per person and month (person-months) by water supply size, sex, age, education level and calendar month in the Norwegian longitudinal cohort study (99,446 monthly submissions; 9,447 participants)

Variables	Number of person-months	
	AGI^a^ (per 100 person-months)	Severe AGI^b^ (per 100 person-months)
	5,508 (5.5)	819 (0.8)
**Size of water supply**		
Small water supplies^c^	1,204 (5.1)	214 (0.9)
Large water supplies^d^	4,304 (5.7)	605 (0.8)
**Tap water drunk**^e^		
0–1 glasses	467 (5,6)	70 (0,8)
2–3 glasses	1392 (5,5)	177 (0,7)
4–5 glasses	1705 (5,2)	249 (0,8)
6–7 glasses	998 (5,8)	157 (0,9)
8 + glasses	946 (5,9)	166 (1)
**Sex**		
Male	2,342 (5)	314 (0,7)
Female	3,166 (6)	505 (1)
**Age**		
0–5	342 (9.5)	33 (0.9)
6–12	221 (4.7)	16 (0.3)
13–18	106 (4.6)	8 (0.3)
19–49	2,066 (7.6)	342 (1.3)
50–64	1,886 (5.3)	302 (0.9)
65–80	887 (3.4)	118 (0.5)
**Education**		
Tertiary education	2,896 (5.7)	445 (0.9)
Primary, secondary, other education	1,964 (5.2)	316 (0.8)
Persons below 18 years	633 (6.3)	56 (0.6)
Missing	15 (4.9)	2 (0.7)
**Region**		
South	85 (5.4)	18 (1.1)
East	3,373 (5.5)	496 (0.8)
West	1,196 (5.6)	167 (0.8)
Middle	421 (5.1)	70 (0.9)
North	433 (5.9)	68 (0.9)
**Calendar-month**		
January	530 (6.5)	83 (1)
February	536 (6.7)	72 (0.9)
March	376 (5.6)	63 (0.9)
April	489 (5.9)	67 (0.8)
May	448 (5.2)	58 (0.7)
June	447 (5.2)	80 (0.9)
July	389 (4.9)	58 (0.7)
August	451 (5.3)	74 (0.9)
September	434 (5.2)	72 (0.9)
October	418 (4.3)	52 (0.5)
November	401 (5.1)	58 (0.7)
December	529 (6.1)	72 (0.8)
Missing	60 (17.3)	10 (2.9)

^a^If the respondent had reported at least one of the following: (i) three or more occurrences of diarrhea or (ii) vomiting the last 24 h

^b^Severe AGI vas defined as five or more occurrences of diarrhea the last 24 h

^c^50–1000 persons supplied

^d^> 1,000 persons supplied

^e^Number of 0.2L glasses (per 24h) per participant

### Crude and adjusted model

The amount of tap water drunk (glasses per day) did not have a statistically significant association with AGI in the adjusted model run on tap water drunk as a continuous variable (*p* = 0.392, Table 4), whereas there was a small statistically significant association in the model run on tap water drunk as a categorical variable (*p* = 0.047, Table 5). The very small risk differences (RD) had nonlinear variation among glasses per day categories and no pairwise comparison was significant when comparing against the baseline of 0–1 glasses per day. Sex was statistically significant in the adjusted models for tap water drunk both as categorical and continuous variable; however, with very small RD estimate (RD = 0.008 (0,004, 0.013), *p* < 0.001, Table 4 and Table 5). Age was statistically significant in the adjusted models (*p* < 0.001, Tables 4 and 5). The RD estimates for those 0–5 years of age were 0.052 (0.017–0.088) in comparison to those for the 19–49 years of age participants (reference group). For the 65- to 80-year-old age group, the RD estimate was -0.043 (-0.049–0.037) (Table 4 and Table 5). There was a statistically significant variation in risk between months (*p* < 0.001, Table 4 and Table 5). The size of the drinking water supply and education level were not significant in the adjusted model.

**Table 4:** Crude and adjusted risk difference (RD) estimates for the continuous amount of tap water drunk (exposure)and monthly acute gastrointestinal infection (AGI) (outcome) from the linear mixed effects models (99,446 monthly submissions; 9,447 participants)

Outcome AGI	Crude		Adjusted	
	Risk difference (95%CI)	***P*** value	Risk difference (RD) (95%CI)	***P*** value
**Tap water drunk**				
Number of 0.2L glasses	0.0005 (-0.0001, 0.001)	0.091	0.0002 (-0.0003, 0.001)	0.392
**Size of water supply**				
Small water supplies^a^	0(Ref)	-	0(Ref)	-
Large water supplies^b^	0.005 (0.0002, 0.011)	0.041	0.004 (-0.001, 0.009)	0.141
**Sex**				
Male	0(Ref)	-	0(Ref)	-
Female	0.011 (0.006, 0.015)	< 0.001	0.008 (0.004, 0.013)	< 0.001
**Age**				< 0.001
19–49 years	0(Ref)	-	0(Ref)	-
0–5 years	0.017 (0.005, 0.029)	0.005	0.052 (0.017, 0.088)	0.004
6–12 years	-0.031 (-0.041,—0.020)	< 0.001	0.004 (-0.031, 0.039)	0.816
13–18 years	-0.032 (-0.047,—0.018)	< 0.001	-0.006 (-0.036, 0.023)	0.683
50–64 years	-0.024 (-0.030, -0.019)	< 0.001	-0.024 (-0.029,—0.018)	< 0.001
65–80 years	-0.045 (-0.051,—0.039)	< 0.001	-0.043 (-0.049, -0.037)	< 0.001
**Education**		0.059		0.101
Tertiary education	0(Ref)	-	0(Ref)	-
Persons below 18 years	0.006 (-0.002, 0.013)	0.149	-0.032 (-0.066, 0.001)	0.057
Primary, secondary, other education	-0.003 (-0.008, 0.001)	0.150	0.002 (-0.003, 0.006)	0.488
**Calendar month**		< 0.001		< 0.001
January	0(Ref)	-	0(Ref)	-
February	0.0002 (-0.006, 0.007)	0.963	0.0001 (-0.006, 0.007)	0.968
March	-0.009 (-0.016, -0.002)	0.009	-0.009 (-0.016, -0.002)	0.010
April	-0.007 (-0.013, -0.0003)	0.040	-0.007 (-0.014, -0.001)	0.035
May	-0.014 (-0.020, -0.008)	< 0.001	-0.014 (-0.021, -0.008)	< 0.001
June	-0.014 (-0.020, -0.007)	< 0.001	-0.014 (-0.020, -0.007)	< 0.001
July	-0.017 (-0.023, -0.010)	< 0.001	-0.017 (-0.023, -0.010)	< 0.001
August	-0.012 (-0.018, -0.005)	< 0.001	-0.012 (-0.018, -0.005)	< 0.001
September	-0.013 (-0.019, -0.006)	< 0.001	-0.013 (-0.019, -0.006)	< 0.001
October	-0.022 (-0.028, -0.015)	< 0.001	-0.022 (-0.028, -0.015)	< 0.001
November	-0.014 (-0.021, -0.008)	< 0.001	-0.014 (-0.021, -0.007)	< 0.001
December	-0.005 (-0.012, 0.001)	0.114	-0.005 (-0.012, 0.001)	0.120

^a^50–1000 persons supplied

^b^> 1000 persons supplied

**Table 5:** Crude and adjusted risk difference (RD) estimates for the categorical amount of tap water drunk (exposure) and monthly acute gastrointestinal infection (AGI) (outcome) from linear mixed effects models (99,446 monthly submissions; 9,447 participants)

Outcome AGI	Crude		Adjusted	
	Risk difference (95%CI)	***P*** value	Risk difference(RD) (95%CI)	***P*** value
**Tap water drunk**				
Number of 0.2L glasses		0.019		0.047
0–1 glasses	0(Ref)	-	0(Ref)	-
2–3 glasses	0.003 (-0.003, 0.009)	0.301	0.003 (-0.002, 0.009)	0.240
4–5 glasses	-0.001 (-0.007, 0.005)	0.719	-0.001 (-0.007, 0.005)	0.806
6–7 glasses	0.005 (-0.001, 0.012)	0.104	0.005 (-0.001, 0.012)	0.122
8 + glasses	0.004 (-0.002, 0.011)	0.205	0.003 (-0.004, 0.009)	0.450
**Size of water supply**				
Small water supplies^a^	0(Ref)	-	0(Ref)	-
Large water supplies^b^	0.005 (0.000, 0.011)	0.041	0.004 (-0.001, 0.009)	0.139
**Sex**				
Male	0(Ref)	-	0(Ref)	-
Female	0.011 (0.006, 0.015)	< 0.001	0.008 (0.004, 0.013)	< 0.001
**Age**		< 0.001		< 0.001
19–49 years	0(Ref)	-	0(Ref)	-
0–5 years	0.017 (0.005, 0.029)	0.005	0.052 (0.017, 0.088)	0.004
6–12 years	-0.031 (-0.041, -0.020)	< 0.001	0.004 (-0.031, 0.039)	0.815
13–18 years	-0.032 (-0.047, -0.018)	< 0.001	-0.006 (-0.036, 0.023)	0.684
50–64 years	-0.024 (-0.030, -0.019)	< 0.001	-0.024 (-0,029, -0.018)	< 0.001
65–80 years	-0.045 (-0.051, -0.039)	< 0.001	-0.043 (-0.049, -0.037)	< 0.001
**Education**		0.059		0.099
Tertiary education	0(Ref)	-	0(Ref)	-
Persons below 18 years	0.006 (-0.002–0.013)	0.149	-0.032 (-0.066–0.001)	0.056
Primary, secondary, other education	-0.003 (-0.008–0.001)	0.150	0.002 (-0.003–0.006)	0.475
**Calendar month**		< 0.001		< 0.001
January	0(Ref)	-	0(Ref)	-
February	0.0002 (-0.006, 0.007)	0.963	0.000 (-0.006, 0.007)	0.969
March	-0.009 (-0.016, -0.002)	0.009	-0.009 (-0.016, -0.002)	0.011
April	-0.007 (-0.013, -0.0003)	0.040	-0.007 (-0.014, -0.0005)	0.035
May	-0.014 (-0.020, -0.008)	< 0.001	-0.014 (-0.021, -0.008)	< 0.001
June	-0.014 (-0.020, -0.007)	< 0.001	-0.014 (-0.020, -0.007)	< 0.001
July	-0.017 (-0.023, -0.010)	< 0.001	-0.017 (-0.023, -0.010)	< 0.001
August	-0.012 (-0.018, -0.005)	< 0.001	-0.012 (-0.018, -0.005)	< 0.001
September	-0.013 (-0.019, -0.006)	< 0.001	-0.013 (-0.019, -0.006)	< 0.001
October	-0.022 (-0.028, -0.015)	< 0.001	-0.022 (-0.028, -0.015)	< 0.001
November	-0.014 (-0.021, -0.008)	< 0.001	-0.014 (-0.021, -0.007)	< 0.001
December	-0.005 (-0.012, 0.001)	0,114	-0.005 (-0.012, 0.001)	0.122

^a^50–1,000 persons supplied

^b^> 1,000 persons supplied

For severe AGI, there was a statistically significant association on tap water drunk as continuous variable in the adjusted model, but RD estimate was very small (RD = 0.0002 (0.00002, 0.0005, *p* = 0.029, Table 6). According to the model for tap water drunk as categorical variable, there was no significant association on the amount of water consumed (Table 7). Sex was statistically significant in the adjusted models for tap water drunk both as categorical and continuous variable; however, the RD estimates were very small (RD = 0.002 (0.001,0.004), *p* < 0.003, Table 6 and Table 7). Age was statistically significant in the adjusted models (*p* < 0.001, Table 6 and Table 7), with very small RD estimates. The calendar months of the response were statistically significant, with small RD estimates varying between months (*p* = 0.029, Table 6 and Table 7). The size of the drinking water supply and education level were not significant in the adjusted model.

**Table 6:** Crude and adjusted risk difference (RD) estimates for the continuous amount of tap water drunk (exposure) and monthly severe acute gastrointestinal infection (severe AGI) (outcome) from linear mixed effects models (99,446 monthly submissions; 9,447 participants)

Outcome Severe AGI	Crude		Adjusted	
	Risk difference (95%CI)	***P*** value	**Risk difference (95%CI)**	***P*** value
**Tap water drunk**				
Number of 0.2L glasses	0.0003 (0.0001, 0.0005)	0.002	0.0002 (0.00002, 0.0005)	0.029
**Size of water supply**				
Small water supplies^a^	0(Ref)	-	0(Ref)	-
Large water supplies^b^	-0.001 (-0.003, 0.0004)	0.139	-0.001 (-0.003, 0.0004)	0.131
**Sex**				
Male	0(Ref)	-	0(Ref)	-
Female	0.003 (0.001, 0.005)	< 0.001	0.002 (0.001, 0.004)	0.003
**Age**				< 0.001
19–49 years	0(Ref)	-	0(Ref)	-
0–5 years	-0.004 (-0.008, 0.001)	0.085	-0.005 (-0.018, 0.007)	0.397
6–12 years	-0.010 (-0.013, -0.006)	< 0.001	-0.011 (-0.024, 0.001)	0.069
13–18 years	-0.009 (-0.015, -0.004)	< 0.001	-0.012 (-0.022, -0.001)	0.029
50–64 years	-0.004 (-0.006, -0.002)	< 0.001	-0.004 (-0.006, -0.002)	< 0.001
65–80 years	-0.008 (-0.010, -0.006)	< 0.001	-0.008 (-0.010, -0.006)	< 0.001
**Education**		0.036		0.649
Tertiary education	0(Ref)	-	0(Ref)	-
Persons below 18 years	-0.003 (-0.006, -0.001)	0.011	0.003 (-0.009, 0.014)	0.673
Primary, secondary, other education	-0.000 (-0.002, 0.001)	0.854	0.001 (-0.001, 0.002)	0.379
**Calendar month**		0.020		0.029
January	0(Ref)	-	0(Ref)	-
February	-0.001 (-0.004, 0.001)	0.375	-0.001 (-0004, 0.002)	0.485
March	-0,001 (-0.004, 0.002)	0.603	-0.0005 (-0.003, 0.002)	0.743
April	-0.002 (-0.005, 0.001)	0.112	-0.002 (-0.005, 0.001)	0.149
May	-0.004 (-0.006, -0.001)	0.008	-0.003 (-0.006, -0.001)	0.012
June	-0.001 (-0.004, 0.002)	0.444	-0.001 (-0.004, 0.002)	0.501
July	-0.003 (-0.006, -0.0002)	0.034	-0.003 (-0.005, 0.00002)	0.052
August	-0.001 (-0.004, 0.001)	0.281	-0.001 (-0.004, 0.001)	0.351
September	-0.002 (-0.004, 0.001)	0.247	-0.001 (-0.004, 0.001)	0.335
October	-0.005 (-0.007, -0.002)	< 0.001	-0.005 (-0.007, -0.002)	< 0.001
November	-0.003 (-0.005, -0.00001)	0.049	-0.002 (-0.005, 0.0003)	0.076
December	-0.002 (-0.005, 0.001)	0.142	-0.002 (-0.004, 0.001)	0.218

^a^50–1,000 persons supplied

^b^> 1,000 persons supplied

**Table 7:** Crude and adjusted risk difference (RD) estimates for the categorical amount of tap water drunk (exposure) and monthly severe acute gastrointestinal infection (severe AGI) (outcome) from linear mixed effects models (99,446 monthly submissions; 9,447 participants)

Outcome Severe AGI	Crude		Adjusted	
	**Risk difference (95%CI)**	***P* value**	**Risk difference (RD) (95%CI)**	***P* value**
**Tap water drunk**				
Number of 0.2L glasses		0.046		0.273
0–1 glasses	0(Ref)	-	0(Ref)	-
2–3 glasses	-0.001 (-0.003, 0.002)	0.549	-0.0004 (-0.003, 0.002)	0.712
4–5 glasses	-0.0003 (-0.003, 0.002)	0.821	-0.0002 (-0.003, 0.002)	0.846
6–7 glasses	0.001 (-0.001, 0.004)	0.312	0.001 (-0.001, 0.004)	0.364
8 + glasses	0.002 (-0.001, 0.005)	0.126	0.001 (-0.001, 0.004)	0.309
**Size of water supply**				
Small water supplies^a^	0(Ref)	-	0(Ref)	-
Large water supplies^b^	-0.001 (-0.003, 0.0004)	0.139	-0.001 (-0.003, 0.0004)	0.130
**Sex**				
Male	0(Ref)	-	0(Ref)	-
Female	0.003 (0.001, 0.005)	< 0.001	0.002 (0.001, 0.004)	0.003
**Age**				< 0.001
19–49 years	0(Ref)	-	0(Ref)	-
0–5 years	-0.004 (-0.008, 0.001)	0.085	-0.005 (-0.018, 0.007)	0.401
6–12 years	-0,010 (-0.013, -0.006)	< 0.001	-0.011 (-0.024, 0.001)	0.070
13–18 years	-0.009 (-0.015, -0.004)	< 0.001	-0.012 (-0.022, -0.001)	0.029
50–64 years	-0.004 (-0.006, -0.002)	< 0.001	-0.004 (-0.006, -0.002)	< 0.001
65–80 years	-0.008 (-0.010, -0.006)	< 0.001	-0.008 (-0.010, -0.006)	< 0.001
**Education**		0.036		0.640
Tertiary education	0(Ref)	-	0(Ref)	-
Persons below 18 years	-0.003 (-0.006, -0.001)	0.011	0.003 (-0.009, 0.014)	0.669
Primary, secondary, other education	-0.000 (-0.002, 0.001)	0.854	0.001 (-0.001, 0.002)	0.371
**Calendar month**		0.020		0.029
January	0(Ref)	-	0(Ref)	-
February	-0.001 (-0.004, 0.001)	0.375	-0.001 (-0.004, 0.002)	0.479
March	-0.001 (-0.004, 0.002)	0.603	-0.0005 (-0.003, 0.002)	0.743
April	-0.002 (-0.005, 0.001)	0.112	-0.002 (-0.005, 0.001)	0.151
May	-0.004 (-0.006, -0.001)	0.008	-0.003 (-0.006, -0.001)	0.013
June	-0.001 (-0.004, 0.002)	0.444	-0.001 (-0.004, 0.002)	0.506
July	-0.003 (-0.006, -0.0002)	0.034	-0.003 (-0.005, -0.000005)	0.050
August	-0.001 (-0.004, 0.001)	0.281	-0.001 (-0.004, 0.001)	0.345
September	-0.002 (-0.004, 0.001)	0.247	-0.001 (-0.004, 0.001)	0.333
October	-0.005 (-0.007, -0.002)	< 0.001	-0.005 (-0.007, -0.002)	< 0.001
November	-0.003 (-0.005, -0.000)	0.049	-0.002 (-0.005, 0.0003)	0.076
December	-0.002 (-0.005, 0.001)	0.142	-0.002 (-0.004, 0.001)	0.216

^a^50–1,000 persons supplied

^b^> 1,000 persons supplied

Extrapolating our data on reported AGI to the entire Norwegian population results in an estimated 3.6 million person-months with AGI per year. Of this, our models estimate that 64,000 (model with continuous glasses of water) and 117,000 (model with categorical glasses of water) person-months are attributable to tap-water consumption, corresponding to approximately 2–4% of AGI in Norway.

## Discussion

In this prospective cohort study investigating AGI among the Norwegian population for a period of 12 months, a total of 9,946 persons participated, for an overall response rate of 11.5%. The cohort participants represented both large and small drinking water supplies, sex (male/female), age, education level and geographical region in Norway.

We found a relatively low number of AGI per 100 person-months (approximately 5), and a very low number of severe AGI per 100 person-months (< 1). The highest number of AGI per 100 person-months was found among those aged 0–5 years (9.5), followed by those aged 19–49 years (7.6). Our study estimates that 2–4% of AGI in Norway is attributable to drinking tap water.

The risk of AGI was higher among small children (0–5 years; 5 percentage points higher risk of AGI compared to those 19–49 years old, corresponding to 0.6 extra AGI-events per year) and lower among the eldest participants (65–80 years; 4 percentage points less than those 19–49 years old, corresponding to 0.5 fewer AGI-events per year). AGI varied by season, and there was also a small association with sex. Education level and size of the drinking water supply were not statistically significant.

The results are not incongruent with those of a previous study in Norway, in which reported an incidence of acute gastroenteritis of 1·2 per person-year [33]. However, the particular study was conducted more than 20 years ago with other methods for data collection, and the current study only allows for one AGI case per person month, which may underestimate the true burden. In addition, several precautionary actions in the drinking water sector have been implemented since the studies were conducted, such as enhanced treatment processes, among others, from a publicly funded program; general improvements in best practices; and updates and revisions of regulations in line with the EU Directive on drinking water. On the other hand, the results of the present study are in line with those of a study in the municipality of Ale, Sweden [39]. The present study included children for whom the Ale-study did not. Children are more susceptible to gastrointestinal infections, which might explain the small association observed.

As an observational study, causality cannot be drawn, and caution must be used when interpreting the results. In the present study, participants were more likely to be female, older, have higher education, and come from the eastern region compared to the general Norwegian population. These differences were accounted for in the adjusted regression analyses. The study did not include the etiology causing the disease. Although adjusted for confounders, viral infection during the winter and bacterial infection during the summer may have affected the outcome, as the 1-year follow-up of the participants was conducted during different time periods across the seasons. Despite the findings of low numbers of AGI among the participants during the study, based on the characteristics of the cohort and adjustment for confounders, we assume that the external validity is high, meaning that the outcome may be generalizable to the population of Norway. However, we might not have captured the extent to which the patients were exposed to contaminated drinking water, as we did not record main breaks or intrusion events during the study and assessed how such events affected the study participants. Contamination events could occur hypothetically at any location in the distribution system, at any time, if three key susceptibility conditions must be met for an accidental intrusion to occur in a distribution system: adverse pressure gradient, intrusion pathway, and contaminant source [45]. Furthermore, the present study was designed to study the overall association between the amount of tap water consumed and AGI incidence, and not linked to period of risk such as after heavy rainfall or floods [46, 47]. Hence, the present study could not capture whether the amount of tap water drunk by the participants was related with risk during such potential high-risk periods. It may be that assessing the risk of AGI associated with the amount of tap water drunk is more relevant to such high-risk periods, rather than overall.

Recall bias, for example, by the participants’ tendency to overestimate their own positive behaviour in retrospect (e.g., drinking a “high and healthy” amount of water) or interest in the topic being studied (e.g., having a motivation to be a part of the study due to a high frequency of disease), may have affected the results. The duration of follow-up was one month in our study. In the Ale-study, a difference between 14-day and 28-day recalls was observed, where shorter recalls were associated with a 20% greater incidence [39]. The study duration was relatively long (12 months), and this, compared to a crisis such as a waterborne outbreak with massive media coverage, may have led the participants to lose interest and leave the study. We were unable to conduct an analysis of the nonresponders. Although mobile phones are a highly common tool among the Norwegian population, because of the easy access to questionnaires via SMS, the response rate was quite low. This has become a common feature among such a data collection method because it is influenced by the massive increase in marked and customer surveys and may also affect the constraints of fulfilling stricter requirements of personal data protection acts.

The very low number of AGI cases associated with amount of tap water drunk may indicate that efforts to safeguard drinking water in Norway, such as regulations, technical improvements, and publicly funded programs, are effective in providing safe drinking water to the public. It can also be assumed that contamination events, either detected by routine monitoring schemes or critical events such as main breaks or similar events, in the distribution system have led to corrective action by the water supplier, such as issuing boil water advisories to the customers of the affected supply area [48–51]. The low incidence of cases underscores the importance of control measures in the drinking water sector, as these measures seem effective. On the other hand, considering the vulnerability of the drinking water distribution system in Norway, it is imperative to continue investing and maintaining the distribution system to avoid future waterborne outbreaks caused by contamination entering the system after water treatment processes [29].

Our study estimates that 2–4% of AGI in Norway is attributable to tap water drunk between 2018 and 2020. During these years, Norway had a stable and relatively robust water supply system, except for a severe waterborne outbreak linked to a contaminated reservoir after heavy rainfall in 2019, which caused approximately 1,500 cases of campylobacteriosis [29]. However, concerns about the risk of waterborne outbreaks are emerging due to an increase in the hygienic load related to the import of new or re-emerging pathogens from the effects of climate change, people travelling abroad, pressure from the expansion of dwelling areas, and activities near raw water sources [30, 31, 52]. With increasing severe weather due to climate change, the quality of water and safe operation of the water supply system in Norway may decrease, limiting the generalizability of these results to the future [53], although documented health effects on waterborne diseases linked to climate change in Norway are scarce [54]. Waterborne outbreaks may have devastating impact on human health [55–58], societal consequences in terms of loss of work days and burden on the health system [59–61], as well as long-term consequences for health [30, 62]. Some of these challenges are common among drinking water supply systems in similar contexts. In a review of waterborne outbreaks in Europe, North America and New Zealand, among 66 identified outbreaks, the causes were the contamination of raw water from surface waters (13/66) and groundwater (11/66), treatment deficiencies in the water treatment plant (18/66) and more than one-third from distribution system failures (26/66) [63]. In terms of outbreaks, it is estimated that in North America, drinking water distribution systems could account for approximately 30% of waterborne outbreaks [64]. The effects of changing climatic factors are expected to act as stressors to aging and vulnerable drinking water supply systems and health consequences [23, 24]. Concern about the ability of small water supply systems to manage a water crisis for effective public health protection is also a concern [26], in which the majority of drinking water supplies in Norway are smaller. Furthermore, other countries may have other challenges, regulations and characteristics related to their water supply systems, limiting the generalizability of these results to other countries.

## Conclusion

This is the largest cohort study in Norway estimating the burden of self-reported gastrointestinal infections linked to the amount of tap water consumed in Norway. Our study estimates that 2–4% of AGI in Norway is attributable to drinking tap water. We found a relatively low number of AGI per 100 person-months (approximately 5), and a very low number of severe AGI per 100 person-months (< 1). There was a small association with age, sex and season. These results may indicate that waterborne AGI is not currently a burden in Norway, although the data should be used with caution. We underline that risks for AGI are present in the drinking water supplies, potentially causing short-term and long-term adverse health consequences in which should be prevented. Aging drinking water distribution systems that are vulnerable to contamination represent a high risk for waterborne outbreaks and sporadic cases of waterborne illness. This emphasises the importance of continued efforts and investments in the maintenance of drinking water supplies in Norway to address the low burden of sporadic waterborne cases and to prevent future outbreaks.

### Supplementary Information

Supplementary Material 1.

## Data Availability

The datasets used and/or analysed during the current study are available from the corresponding author on a reasonable request.
