# Supplementary material for: Estimating the risk of gastrointestinal illness associated with drinking tap water in Norway: a prospective cohort study
Source: BMC Public Health. 2024 Aug 5;24:2107. doi: 10.1186/s12889-024-19607-2 (PMC11299283; doi:10.1186/s12889-024-19607-2)
Supplement: Supplementary file 1 — Supplementary Material 1. [file 12889_2024_19607_MOESM1_ESM.docx]

# Supplementary material

Initial recruitment interviews and monthly surveys developed in the study “Estimating the risk of gastrointestinal illness associated with drinking water in Norway: a prospective cohort study” are here translated from Norwegian to English.

The initial recruitment interview and the ending interview after the study period was over are similar. Note that the questions are not chronological as they followed an index depending on the answers on certain questions.

## Initial interviews

**Initial interview by electronic survey Drinking Water Study Participants 16 years and above**

Q001

Welcome to the Drinking Water Study, a nationwide research project aimed at determining how many people in Norway become ill from drinking water. Only transient gastrointestinal illness (diarrhea and/or vomiting) is being investigated. Other illnesses are not studied in this research.

CONSENT FORM

According to government guidelines, we ask you to initially confirm the following:

By participating in the study, you will be asked to answer two questionnaires; one before the study starts and one when the study is completed. In addition, once a month for twelve months, you will receive an SMS with two questions about how much water you have drunk in the last 24 hours and whether you have had gastrointestinal illness in the last month. If you have been ill, you will be asked about the symptoms you experienced and how long they lasted. If you wish to learn more about the study, you can visit our website [www.fhi.no/studier/drikkevannsstudien](http://www.fhi.no/studier/drikkevannsstudien).

Participation in the study is voluntary, and you can withdraw from the study at any time without giving a reason. You can also request that collected information about you be deleted. If you have any questions about the study, you can contact us at [www.fhi.no/studier/drikkevannsstudien/kontakt](http://www.fhi.no/studier/drikkevannsstudien/kontakt), and if you have technical problems, you can contact Norwegian Gallup at 800 84 700.

All information will be anonymized and deleted no later than five years after the end of the project. The study is approved by the Regional Committees for Medical and Health Research Ethics, REK (2016/1422).

- Yes, I have read and understood the information above, and I want to participate in the study
- No, I do not want to participate in the study

Q002

Your household How many people are there in the household where you live, including yourself?

1. 1 (living alone)
2. 2
3. 3-5
4. 6 or more
5. Don't know

Ask only if Q002,2,3,4,5

Q003

Are any of these people children under seven years old?

1. Yes
2. No
3. Don't know

Ask only if Q003,1

Q004

Does any of the children attend daycare?

1. Yes
2. No
3. Don't know

Q005

What type of housing do you live in?

1. Farm with livestock
2. Detached house
3. Semi-detached house
4. Townhouse/Row house
5. Apartment building
6. Other type of residence
7. Other
8. Don't know

Q006

Do you have pets in your household? Check all that apply.

1. Dog
2. Cat
3. Pet bird
4. Rabbit
5. Guinea pig
6. Hamster
7. Mouse/rat
8. Aquarium fish
9. Turtle
10. Lizard/snake
11. Other pet
12. No, no pets
13. Don't know

Q007

Do you have weekly contact with farm animals? Check all that apply.

1. Cattle
2. Pig
3. Sheep
4. Goat
5. Horse
6. Poultry
7. Reindeer
8. Other farm animal
9. No, no weekly contact with any of these animals
10. Don't know

Q025

Do you have contact with wild animals and birds? Check all that apply.

1. Feed small birds in the garden
2. Feed hedgehogs in the garden
3. Contact with other animals
4. No, I do not have contact with wild animals and birds
5. Don't know

Q008

What is your highest completed education?

1. Did not complete primary school
2. Lower secondary school, primary school, middle school
3. Upper secondary school, high school, vocational training, etc.
4. University or college
5. Other education
6. Don't know

Q025

Do you study / work in the same municipality as you live in?

1. Yes
2. No
3. Not relevant (not working/studying)
4. Don't know

Q009

Diet and kitchen hygiene

How often do you eat...

|  | **Always** | **Usually** | **Sometimes** | **Rarely** | **Never** | **Not applicable** | **Don't know** |
| --- | --- | --- | --- | --- | --- | --- | --- |
| ... eat food prepared on a grill or campfire, in the summer season? |  |  |  |  |  |  |  |
| ... eat food from a restaurant, cafeteria, canteen, or other large kitchen? |  |  |  |  |  |  |  |
| ... poultry (chicken, turkey)? |  |  |  |  |  |  |  |
| ... red meat (beef, pork, lamb)? |  |  |  |  |  |  |  |
| ... fish and/or shellfish? |  |  |  |  |  |  |  |
| ... vegetables and/or salad? |  |  |  |  |  |  |  |
| ... fruit and/or berries? |  |  |  |  |  |  |  |

Q026

How often do you wash...

|  | **Always** | **Usually** | **Sometimes** | **Rarely** | **Never** | **Not relevant** | **Don't know** |
| --- | --- | --- | --- | --- | --- | --- | --- |
| ... hands before you eat |  |  |  |  |  |  |  |
| ... hands before you prepare food |  |  |  |  |  |  |  |
| ... knives and other kitchen utensils, after contact with raw meat and before use with other foods (e.g., vegetables or salad) |  |  |  |  |  |  |  |

Q010

Water consumption

How many glasses of tap water do you usually drink per day? Answer with a number; 0,1,2,3,4,5,6, etc. Include ALL water at home, at work, school, exercise, visits, etc. – except bottled water and boiled water. Also include water used to make juice or ice cubes. 1 glass = 2 dl. If no glasses, write 0.

Ask only if Q010 >= 1

Q011

How many glasses of tap water do you usually drink at home per day? Answer with a number; 0,1,2,3,4,5,6, etc. Include water used to make juice or ice cubes, but NOT bottled water or boiled water. 1 glass = 2 dl. If no glasses, write 0.

Ask only if Q010 >= 1 and NOT Q025,4

Q014

Do you drink water directly from a river, stream, or lake when hiking or in the forest?

1. Yes
2. No
3. Do not go hiking
4. Don't know

Q015

Do you have access to a cabin / vacation home?

1. Yes
2. No
3. Don't know

Ask only if Q015,1

Q016

How many times did you go to the cabin in the last 12 months? Answer with a number; 0,1,2,3,4, etc. If none, write 0. If multiple cabins/vacation homes, answer for the one most frequently used in the last 12 months.

Ask only if Q015,1

Q017

Where does the drinking water at the cabin/vacation home mainly come from? If multiple cabins/vacation homes, answer for the one most frequently used in the last 12 months.

1. Brought water
2. Private water source / well
3. Common water supply
4. Other, please specify:
5. Don't know

Q021

Acute gastrointestinal infection

How many times have you had gastrointestinal illness in the last twelve months? Gastrointestinal illness is sudden loose stools either alone or together with nausea, vomiting, stomach pain, and/or fever. Answer with a number; 0,1,2,3, etc., where 0 means you have not had gastrointestinal illness.

Ask only if Q021 >= 1

Q022

Did you contact a doctor?

1. Yes
2. No
3. Don't know

Q024

Thank you very much for your participation! You will now receive one SMS per month for twelve months, with a link to a short questionnaire about how much water you have drunk and whether you have had gastrointestinal illness in the last month.

**Initial Interview by electronic survey Drinking Water Study Participants Under 16 Years**

Q001

Welcome to the Drinking Water Study, a nationwide research project aimed at determining how many people in Norway get sick from drinking water. Only temporary stomach illnesses (diarrhea and/or vomiting) are being investigated in this study. Other illnesses are not being examined in this study.

The form is filled out on behalf of the child in the household selected to participate in the study, hereinafter referred to as "the child".

Q002

Household

How many people are there in the household where the child lives?

1. 2
2. 3-5
3. 6 or more
4. Don't know

Q003

Is the child under seven years old?

1. Yes
2. No
3. Don't know

Q004

Does the child attend daycare?

1. Yes
2. No
3. Don't know

Q005

Are there other children under seven years old in the household, besides the child participating in the survey?

1. Yes
2. No
3. Don't know

Q006

Do any of these other children attend daycare?

1. Yes
2. No
3. Don't know

Q007

What type of housing does the child live in?

1. Farm with livestock
2. Detached house
3. Semi-detached house
4. Rowhouse/Attached house
5. Apartment building
6. Other type of housing
7. Other
8. Don't know

Q008

Are there pets in the household? Check all that apply

1. Dog
2. Cat
3. Caged bird
4. Rabbit
5. Guinea pig
6. Hamster
7. Mouse/rat
8. Aquarium fish
9. Turtle
10. Lizard/snake
11. Other pet
12. No, no pets
13. Don't know

Q009

Does the child have weekly contact with farm animals? Check all that apply

1. Cattle
2. Pig
3. Sheep
4. Goat
5. Horse
6. Poultry
7. Reindeer
8. Other farm animals
9. No, no weekly contact with farm animals
10. Don't know

Q010

Does the child have contact with wild animals and birds? Check all that apply

1. Feeds small birds in the garden
2. Feeds hedgehogs in the garden
3. Contact with other animals
4. No, no contact with wild animals and birds
5. Don't know

Q011

Diet and Kitchen Hygiene

How often does the child...

|  | **Always** | **Usually** | **Sometimes** | **Rarely** | **Never** | **Not applicable** | **Don't know** |
| --- | --- | --- | --- | --- | --- | --- | --- |
| ... eat food prepared on a grill or campfire, in the summer season? |  |  |  |  |  |  |  |
| ... eat food from a restaurant, cafeteria, canteen, or other large kitchen? |  |  |  |  |  |  |  |
| ... poultry (chicken, turkey)? |  |  |  |  |  |  |  |
| ... red meat (beef, pork, lamb)? |  |  |  |  |  |  |  |
| ... fish and/or shellfish? |  |  |  |  |  |  |  |
| ... vegetables and/or salad? |  |  |  |  |  |  |  |
| ... fruit and/or berries? |  |  |  |  |  |  |  |

Q019

How often does the child wash...

|  | **Always** | **Usually** | **Sometimes** | **Rarely** | **Never** | **Not relevant** | **Don't know** |
| --- | --- | --- | --- | --- | --- | --- | --- |
| ... hands before he/she eats |  |  |  |  |  |  |  |
| ... hands before he/she prepares food |  |  |  |  |  |  |  |
| ... knives and other kitchen utensils, after contact with raw meat and before use with other foods (e.g., vegetables or salad) |  |  |  |  |  |  |  |

Q012

Water Consumption

How many glasses of tap water does the child usually drink per day?

Answer with numbers; 0,1,2,3,4,5,6, etc. Include ALL water (at home, at school, in daycare, in recreational activities, visits, etc.) – except purchased bottled water and boiled water. Also include water used to make juice or ice cubes. 1 glass = 2 dl. If no glasses, write 0 (zero).

Q013

How many glasses of tap water does the child usually drink at home, where he/she lives, per day?

Answer with numbers; 0,1,2,3,4,5,6, etc. Include water used to make juice or ice cubes, but NOT bottled water or boiled water. 1 glass = 2 dl. If no glasses, write 0 (zero).

Ask only if Q012 >= 1

Q014

How many glasses of tap water does the child usually drink in daycare / at school per day?

Answer with numbers; 0,1,2,3,4,5,6, etc. Include water used to make juice or ice cubes, but NOT bottled water or boiled water. 1 glass = 2 dl. If no glasses, write 0 (zero). If the child is not in school/daycare, check "Not relevant".

Q015

Does the child drink water directly from a river, stream, or lake when he/she is on a mountain or forest trip?

1. Yes
2. No
3. Does not go on trips
4. Don't know

Q016

Do you have access to a cabin / recreational property?

1. Yes
2. No
3. Don't know

Ask only if Q016,1

Q017

How many times has the child been to the cabin / recreational property in the last twelve months?

Answer with numbers; 1,2,3,4, etc. If none, write 0 (zero). If multiple cabins/recreational properties, answer for the one most frequently used in the last 12 months.

Q018

Where does the drinking water at the cabin/recreational property mainly come from?

If multiple cabins/recreational properties, answer for the one most frequently used in the last 12 months.

1. Brought water
2. Private water source/well
3. Public water supply
4. Other, please specify:
5. Don't know

Q022

Acute gastrointestinal infection (stomach upset)

How many times has the child had a stomach illness in the last twelve months?

Stomach illness is sudden loose stools either alone or along with nausea, vomiting, stomach pain, and/or fever. Answer with numbers; 0,1,2,3, etc., where 0 means the child has not had a stomach illness.

Ask only if Q022 >= 1

Q023

Was a doctor contacted?

1. Yes
2. No
3. Don't know

Q024

Thank you for participating! You will now receive one SMS per month for twelve months with a link to a short questionnaire about how much water the child has drunk and whether he/she has had a stomach illness in the last month.

## Monthly surveys

**Monthly SMS-questionnaire Drinking water study 16 years and older**

Q015

Hello, here is your monthly questionnaire in the Drinking Water Study, conducted by the Norwegian Institute of Public Health.

Q001

How many glasses of tap water (Note! Not bottled water or boiled water) have you drunk in the last 24 hours?

Respond with numbers; 0,1,2,3,4,5,6, etc. 1 glass = 2 dl. If no glasses, write 0 (zero). Include ALL water you have drunk in the last day (at home, at work, school, exercise, visits, etc.) - including water used to make juice or ice cubes.

Q002

How many times have you been sick with stomach problems in the last 28 days?

Stomach problems are sudden loose stools either alone or accompanied by nausea, vomiting, stomach pain, and/or fever. Respond with numbers; 0,1,2,3, etc., where 0 means you have not been sick with stomach problems.

Scripter notes: Don't know in numerical questions can be entered as a separate checkbox. Also applies to the following. Answer 0 (zero) and Don't know: END INTERVIEW

Ask only if Q002 == 1

B001: SICK AFTER WATER CONSUMPTION - ONE OCCURRENCE

Q003

You will now be asked a few questions about symptoms and duration.

Q006

Where did you drink water during the last two weeks before you were sick?

Here we want you to include all water, whether it is tap water or water directly from nature (but not boiled water/bottled water). Check all that apply.

1. At home
2. At work/study place
3. At the cabin 4 Directly from river, stream, or lake

5 Other place

6 Don't know

Q005

Have you travelled abroad (at least one overnight stay outside Norway) in the last two weeks before you became sick?

1. Yes
2. No
3. Don't know

Q016

Which of the following symptoms did you have when you were sick?

Check all that apply.

1. Diarrhea
2. Vomiting
3. Nausea
4. Stomach ache
5. Fever
6. Don't know

Ask only if Q016,1

Q017

How many times did you have diarrhea in one day at most, when you were sick?

Respond with numbers; 0,1,2,3, etc., where 0 means no diarrhea episodes.

Q018

How many days did your illness last when you were sick?

Write with numbers the number of days. Round up, if you were sick for 1.5 days, write 2.

Q004

Did you contact a doctor when you were sick?

1. Yes
2. No
3. Don't know

Q032

How many days of absence (from work and/or study place) did you have when you were sick?

Respond with numbers; 0,1,2,3, etc., where 0 means you did not have any absences. Round up, if you were absent from work and/or study place for 1.5 days, write 2. If you do not work or study, check "Not applicable."

Q033

Were you hospitalized in connection with being sick?

1. Yes
2. No
3. Don't know

Ask only if Q033,1

Q034

How many days did the hospital stay last when you were sick?

Respond with numbers; 0,1,2,3, etc., where 0 means you were not hospitalized. Round up, if you were hospitalized for 1.5 days, write 2.

Ask only if Q002 >= 2

MULTIPLE OCCURRENCES - EPISODE 1

Q007

You will now be asked some questions about symptoms and duration of your cases of stomach problems. The first questions apply to the first time you were sick in the last 28 days.

Q021

Where did you drink water during the last two weeks before the first time you were sick?

Here we want you to include all water, whether it is tap water or water directly from nature (but not boiled water/bottled water). Check all that apply.

1. At home
2. At work/study place
3. At the cabin
4. Directly from river, stream, or lake
5. Other place
6. Don't know

Q020

Have you travelled abroad (at least one overnight stay outside Norway) in the last two weeks before you became sick for the first time?

1. Yes
2. No
3. Don't know

Q008

Which of the following symptoms did you have the first time you were sick?

Check all that apply.

1. Diarrhea
2. Vomiting
3. Nausea
4. Stomach ache
5. Fever
6. Don't know

Ask only if Q008,1

Q009

How many times did you have diarrhea in one day at most the first time you were sick?

Respond with numbers.

Q010

How many days did your illness last the first time you were sick?

Respond with numbers for the number of days. Round up, if you were sick for 1.5 days, write 2.

Q019

Did you contact a doctor the first time you were sick?

1. Yes
2. No
3. Don't know

Q026

How many days of absence (from work and/or study place) did you have the first time you were sick?

Respond with numbers; 0,1,2,3, etc., where 0 means you did not have any absences. Round up, if you were absent from work and/or study place for 1.5 days, write 2. If you do not work or study, check "Not applicable."

Q027

Were you hospitalized in connection with being sick for the first time?

Q028

How many days did the hospital stay last the first time you were sick?

Respond with numbers; 0,1,2,3, etc., where 0 means you were not hospitalized. Round up, if you were hospitalized for 1.5 days, write 2.

Ask only if Q002 == 2

MULTIPLE OCCURRENCES - EPISODE 2

Q025

Now we will ask you some questions about when you were sick the second time.

Q024

Where did you drink water during the last two weeks before you were sick the second time?

Here we want you to include all water, whether it is tap water or water directly from nature (but not boiled water/bottled water). Check all that apply.

1. At home
2. At work/study place
3. At the cabin
4. Other place
5. Don't know

Q023

Have you travelled abroad (at least one overnight stay outside Norway) in the last two weeks before you became sick for the second time?

1. Yes
2. No
3. Don't know

Q011

Which of the following symptoms did you have the second time you were sick?

1. Diarrhea
2. Vomiting
3. Nausea
4. Stomach ache
5. Fever
6. Don't know

Ask only if Q011,1

Q012

How many times did you have diarrhea in one day at most, the second time you were sick?

Write the number of times.

Q013

How many days did your illness last, the second time you were sick?

Write the number of days - round up (e.g., for 1.5 days, write 2).

Q022

Did you contact a doctor the second time you were sick?

1. Yes
2. No
3. Don't know

Q029

How many days of absence (from work and/or study place) did you have the second time you were sick?

Respond with numbers; 0,1,2,3, etc., where 0 means you did not have any absences. Round up, if you were absent from work and/or study place for 1.5 days, write 2. If you do not work or study, check "Not applicable."

Q030

Were you hospitalized in connection with being sick the second time?

1. Yes
2. No
3. Don't know

Ask only if Q030,1

Q031

How many days did the hospital stay last the second time you were sick?

Respond with numbers; 0,1,2,3, etc., where 0 means you were not hospitalized. Round up, if you were hospitalized for 1.5 days, write 2.

Q014

Thank you for participating! We will be back in a month. Have a nice day.

**Monthly SMS-questionnaire Drinking Water Study participants below 16 years**

Q1

Hello, here is the monthly questionnaire for the Drinking Water Study, conducted by the Norwegian Institute of Public Health.

The form is filled out on behalf of the child in the household selected to participate in the study, hereinafter referred to as "the child."

Q2

How many glasses of tap water (NB! Not bottled water or boiled water) has the child drunk in the last 24 hours?

Respond with numbers; 0, 1, 2, 3, 4, 5, 6, etc., 1 glass = 2 dl. If no glasses, write 0 (zero). Include ALL water the child has consumed in the last day (at home, in daycare, at school, during extracurricular activities, visits, etc. - including water used to make juice or ice cubes).

Q3

How many times has the child had stomach illness in the last 28 days? Stomach illness is sudden loose stools either alone or along with nausea, vomiting, stomach pain, and/or fever. Respond with numbers; 0, 1, 2, 3, etc., where 0 means the child has not had stomach illness. Scripter notes: "Don't know" in numerical questions can be included as a separate checkbox. Also applies to the following. Answer 0 (zero) and Don't know: END INTERVIEW

Ask only if Q003x == 1 ILLNESS AFTER WATER CONSUMPTION

Q4

You will now receive a few questions about symptoms and duration.

Q5 Where did the child drink water during the last two weeks before the illness started when she/he was sick? Here we want you to include all water, whether it is tap water or water directly from nature (but not boiled water/bottled water). Check all that apply.

1. At home
2. At school/In daycare
3. At the cabin
4. Directly from river, stream, or lake
5. Other place
6. Don't know

Q6

Was the child on an overseas trip (at least one overnight stay outside Norway) in the last two weeks before she/he became sick?

1. Yes
2. No
3. Don't know

Q7

Which of the following symptoms did the child have when she/he was sick?

Check all that apply.

1. Diarrhea
2. Vomiting
3. Nausea
4. Stomach ache
5. Fever
6. Don't know

Ask only if Q007,1

Q8

How many times did the child have diarrhea in one day at most when she/he was sick? Respond with numbers, 0, 1, 2, 3, etc., where 0 means no diarrhea episodes.

Q9

How many whole days was the child sick after she/he became sick?

Respond with numbers for the number of days. Round up, if the child was sick for 1.5 days, write 2.

Q10

Was a doctor contacted when the child was sick?

1. Yes
2. No
3. Don't know

Q11

How many days of absence (from daycare/school) did the child have when she/he was sick?

Respond with numbers; 0, 1, 2, 3, etc., where 0 means the child did not have any absences. Round up, if the child was absent from daycare/school for 1.5 days, write 2.

Q12

Was the child hospitalized in connection with being sick?

1. Yes
2. No
3. Don't know

Q13

How many days did the hospital stay last? Respond with numbers; 0, 1, 2, 3, etc., where 0 means the child was not hospitalized. Round up, if the child was hospitalized for 1.5 days, write 2.

Ask only if Q003 >= 2 MULTIPLE OCCURRENCES - EPISODE 1

Q14

We will now ask a few questions about the illness episodes, starting with the first one.

Q15

FIRST TIME THE CHILD WAS SICK: Where did the child drink water during the last two weeks before the illness started when she/he was sick for the first time? Here we want you to include all water, whether it is tap water or water directly from nature (but not boiled water/bottled water). Check all that apply.

1. At home
2. At school/In daycare
3. At the cabin
4. Directly from river, stream, or lake
5. Other place
6. Don't know

Q16

FIRST TIME THE CHILD WAS SICK: Was the child on an overseas trip (at least one overnight stay outside Norway) in the last two weeks before she/he became sick for the first time?

1. Yes
2. No
3. Don't know

Q17

FIRST TIME THE CHILD WAS SICK: Which of the following symptoms did the child have the first time she/he was sick? Check all that apply.

1. Diarrhea
2. Vomiting
3. Nausea
4. Stomach ache
5. Fever
6. Don't know

Q18

FIRST TIME THE CHILD WAS SICK: How many times did the child have diarrhea in one day at most, when she/he was sick for the first time? Respond with numbers; 0,1,2,3, etc., where 0 means no diarrhea episodes.

Q19

FIRST TIME THE CHILD WAS SICK: How many whole days was the child sick after she/he became sick for the first time? Respond with numbers for the number of days. Round up, if the child was sick for 1.5 days, write 2.

Q20

FIRST TIME THE CHILD WAS SICK: Was a doctor contacted when the child was sick for the first time?

1. Yes
2. No
3. Don't know

Q21

FIRST TIME THE CHILD WAS SICK: How many days of absence (from daycare/school) did the child have when she/he was sick for the first time? Respond with numbers; 0, 1, 2, 3, etc., where 0 means the child did not have any absences. Round up, if the child was absent from daycare/school for 1.5 days, write 2. If the child is not in daycare/school, check "Not applicable."

Q22

FIRST TIME THE CHILD WAS SICK: Was the child hospitalized in connection with being sick for the first time?

1. Yes
2. No
3. Don't know

Q23

FIRST TIME THE CHILD WAS SICK: How many days did the hospital stay last? Respond with numbers; 0, 1, 2, 3, etc., where 0 means the child was not hospitalized. Round up, if the child was hospitalized for 1.5 days, write 2.

Ask only if Q003 == 2 MULTIPLE OCCURRENCES - EPISODE 2

Q24

You will now receive questions about the second time the child was sick in the last 28 days.

Q25

SECOND TIME THE CHILD WAS SICK: Where did the child drink water during the last two weeks before the illness started when she/he was sick for the second time? Here we want you to include all water, whether it is tap water or water directly from nature (but not boiled water/bottled water). Check all that apply.

1. At home
2. At school/In daycare
3. At the cabin
4. Directly from river, stream, or lake
5. Other place
6. Don't know

Q26

SECOND TIME THE CHILD WAS SICK: Was the child on an overseas trip (at least one overnight stay outside Norway) in the last two weeks before she/he became sick for the second time?

1. Yes
2. No
3. Don't know

Q27

SECOND TIME THE CHILD WAS SICK: Which of the following symptoms did the child have the second time she/he was sick?

Check all that apply.

1. Diarrhea
2. Vomiting
3. Nausea
4. Stomach ache
5. Fever
6. Don't know

Ask only if Q027,1

Q28

SECOND TIME THE CHILD WAS SICK: How many times did the child have diarrhea in one day at most, when she/he was sick for the second time? Respond with numbers; 0,1,2,3, etc., where 0 means no diarrhea episodes.

Q29

SECOND TIME THE CHILD WAS SICK: How many whole days was the child sick after she/he became sick for the second time? Respond with numbers for the number of days. Round up, if the child was sick for 1.5 days, write 2.

Q30

SECOND TIME THE CHILD WAS SICK: Was a doctor contacted when the child was sick for the second time?

1. Yes
2. No
3. Don't know

Q31

SECOND TIME THE CHILD WAS SICK: How many days of absence (from daycare/school) did the child have when she/he was sick for the second time? Respond with numbers; 0, 1, 2, 3, etc., where 0 means the child did not have any absences. Round up, if the child was absent from daycare/school for 1.5 days, write 2. If the child is not in daycare/school, check "Not applicable."

Q32

SECOND TIME THE CHILD WAS SICK: Was the child hospitalized in connection with being sick for the second time?

1. Yes
2. No
3. Don't know

Ask only if Q032,1

Q33

SECOND TIME THE CHILD WAS SICK: How many days did the hospital stay last? Respond with numbers; 0, 1, 2, 3, etc., where 0 means the child was not hospitalized. Round up, if the child was hospitalized for 1.5 days, write 2.

Q34

Thank you very much - that's all. We'll be back in a month. Have a great day.

**Supporting Information**

1. **Information collected from interview & monthly questionnaire**

**Table S1** Information collected per participant by start-up and final questionnaire (e-survey) in the longitudinal cohort study estimating the risk of gastrointestinal illness associated with drinking water in Norway.

| **Type of variable** | **Example of questions/information collected** |
| --- | --- |
| Participant | A unique number per participant, age, sex, and place of residence (county and municipality) |
| Date | Start-up interview date |
| Household | Number of persons in household, children in daycare |
| Education | Higher education, secondary education, primary education or other |
| Farmed animals | Contact with farmed animals such as cattle, pig, sheep, goat, horse, poultry, reindeer or other |
| Pets/Animals | Contact with pets/animals such as…. |
| Hygiene | Frequency of washing hands before cooking, washing kitchen equipment and washing hands before eating. The answers were converted to a number between 0 and 4, and the average of this was used as hygienic score |
| Water consumption | Total glasses and place of water consumption (at home, at work/studies or in nature) |
| Gastrointestinal illness | Number of AGI episodes last 12 months |
| Waterwork | Supplying waterwork size |

**Table S2** Information collected per participant and month (SMS-based questionnaires) in the longitudinal cohort study estimating the risk of gastrointestinal illness associated with drinking water in Norway.

| **Type of variable** | **Example of information collected** |
| --- | --- |
| Participant | A unique number per participant to link with information in start-up interview |
| Date | Date of dispatch of form, date when participant submitted the form, used to defined season |
| Number | Serial number for dispatch of questionnaire |
| Water consumption | Total glasses, drank water at home, at work/place of study, cabin or nature |
| Travel | Abroad or domestic (only answered of those with AGI) |
| Clinical signs | Diarrhea, vomiting, nausea, stomach pain, fever, toilet visits, duration of symptoms, contacted doctor, days in absence from work, hospitalized (duration) |
| Gastrointestinal illnes | AGI last 28 days |

1. **Norway population data**

**Table S3** Norway population by age (1 Jan 2019)^1^.

| **Age** | **Population** |
| --- | --- |
| 0-5 | 356139 |
| 6-12 | 452818 |
| 13-18 | 379912 |
| 19-49 | 2225015 |
| 50-64 | 995487 |
| 65-80 | 718991 |
| 81+ | 199850 |
| Total | 5328212 |

^1^Source: Statistics Norway (<https://www.ssb.no/en>)

**Table S4** Norway population by region (1 Jan 2019)^1^ **.**

| **Region^2^** | **0-80 year** | **81 and older** | **Total** |
| --- | --- | --- | --- |
| South | 294062 | 11182 | 305244 |
| East | 2598015 | 99126 | 2697141 |
| West | 1323113 | 52202 | 1375315 |
| Middle | 446503 | 17557 | 464060 |
| North | 466669 | 19783 | 486452 |
| Norway | 5128362 | 199850 | 5328212 |

^1^ Source: Statistics Norway, <https://www.ssb.no/en>

^2^ **South:** County of Aust-Agder and Vest-Agder; **East:** County of Østfold, Akershus, Oslo, Hedmark, Oppland, Buskerud, Vestfold and Telemark; **West:** County of Rogaland, Hordaland, Sogn-og-fjordane and Møre and Romsdal; **Middle:** County of Trøndelag; **North:** County of Nordland, Troms and Finnmark.

**Table S5** Norway population by region and county (1 Jan 2019)^1^.

| **Region** | **County** | **0-80 year** | **81 and older** | **Total** |
| --- | --- | --- | --- | --- |
| South | Aust-Agder | 113311 | 4344 | 117655 |
|  | Vest-Agder | 180751 | 6838 | 187589 |
| East | Østfold | 285489 | 12031 | 297520 |
|  | Akershus | 603229 | 20826 | 624055 |
|  | Oslo | 662352 | 18719 | 681071 |
|  | Hedmark | 187919 | 9487 | 197406 |
|  | Oppland | 180616 | 8929 | 189545 |
|  | Buskerud | 272091 | 11057 | 283148 |
|  | Vestfold | 240624 | 10454 | 251078 |
|  | Telemark | 165695 | 7623 | 173318 |
| West | Rogaland | 460748 | 14906 | 475654 |
|  | Hordaland | 504454 | 20041 | 524495 |
|  | Sogn-og-fjordane | 104349 | 5425 | 109774 |
|  | Møre og Romsdal | 253562 | 11830 | 265392 |
| Middle | Trøndelag | 446503 | 17557 | 464060 |
| North | Nordland | 232397 | 10988 | 243385 |
|  | Troms | 161087 | 6115 | 167202 |
|  | Finnmark | 73185 | 2680 | 75865 |
| Norway |  | 5128362 | 199850 | 5328212 |

^1^ Source: Statistics Norway (<https://www.ssb.no/en>)

1. **Invitation and recruitmemt**

**Table S6** Invited and recruited by age.

| Age | Population  (1 Jan 2019) | Invited  (% of population) | Participants  (% of invited) | Participants  included  in analysis  (% of invited) | Participants  excluded  from  analysis |
| --- | --- | --- | --- | --- | --- |
| 00-05 | 356139 | 4567 (1.3%) | 352 (7.7%) | 347 (7.6%) | 5 |
| 06-12 | 452818 | 5786 (1.3%) | 490 (8.5%) | 469 (8.1%) | 21 |
| 13-18 | 379912 | 3958 (1.0%) | 267 (6.7%) | 248 (6.3%) | 19 |
| 19-29 | 779007 | 7886 (1.0%) | 441 (5.6%) | 400 (5.1%) | 41 |
| 30-49 | 1446008 | 20633 (1.4%) | 2500 (12.1%) | 2344 (11.4%) | 156 |
| 50-64 | 995487 | 22864 (2.3%) | 3414 (14.9%) | 3274 (14.3%) | 140 |
| 65-80 | 718991 | 20532 (2.9%) | 2490 (12.1%) | 2365 (11.5%) | 125 |
| Total | 5328212 | 86226 (1.6%) | 9954 (11.5%) | 9447 (11.0%) | 507 |

**Table S7** Invited and recruited by region.

| Region | Population  (1 Jan 2019) | Invited  (% of population) | Participants  (% of invited) | Participants  included  in analysis  (% of invited)) | Participants  excluded  from  analysis |
| --- | --- | --- | --- | --- | --- |
| South | 294062 | 2089 (0.7%) | 155 (7.4%) | 151 (7.2%) | 4 |
| East | 2598015 | 45762 (1.8%) | 6102 (13.3%) | 5774 (12.6%) | 328 |
| West | 1323113 | 20348 (1.5%) | 2156 (10.6%) | 2046 (10.1%) | 110 |
| Middle | 446503 | 7786 (1.7%) | 806 (10.4%) | 778 (10.0%) | 28 |
| North | 466669 | 10241 (2.2%) | 735 (7.2%) | 698 (6.8%) | 37 |
| Norway | 5128362 | 86226 (1.7%) | 9954 (11.5%) | 9447 (11.0%) | 507 |

^1^ Source: Statistics Norway (<https://www.ssb.no/en>)

^2^ ^2^**South:** County of Aust-Agder and Vest-Agder; **East:** County of Østfold, Akershus, Oslo, Hedmark, Oppland, Buskerud, Vestfold and Telemark; **West:** County of Rogaland, Hordaland, Sogn-og-fjordane and Møre and Romsdal; **Middle:** County of Trøndelag; **North:** County of Nordland, Troms and Finnmark.

**Table S8** Invited and recruited by waterwork category (persons supplied)

| Waterwork size (persons supplied) | Total number of water-works in Norway | Invited water-works | Participating waterworks | Participating waterworks  included  in analysis | Participants | Participants  (% of invited) | Participants  included  in analysis  (% of invited) | Participants  excluded  from  analysis |
| --- | --- | --- | --- | --- | --- | --- | --- | --- |
| <1000 | 1185 | 374 | 328 | 325 | 26559 | 2352 (8.9%) | 2224 (8.4%) | 128 |
| 1000-4999 | 250 | 31 | 31 | 31 | 14417 | 1388 (9.6%) | 1338 (9.3%) | 50 |
| 5000-19999 | 110 | 10 | 10 | 10 | 17615 | 1977 (11.2%) | 1865 (10.6%) | 112 |
| 20000-100000 | 51 | 5 | 5 | 5 | 13817 | 2023 (14.6%) | 1903 (13.8%) | 120 |
| >100000 | 5 | 5 | 5 | 5 | 13818 | 2214 (16.0%) | 2117 (15.3%) | 97 |
| Total | 1601 | 425 | 379 | 376 | 86226 | 9954 (11.5%) | 9447 (11.0%) | 507 |

1. **Characteristics of the participants**

**Table S8.** Characteristics of the participants (one per household) in the Norwegian longitudinal cohort study divided by small (50-1000 persons supplied) and large (>=1000 persons supplied) waterworks.

| Variable | Category | Small waterworks | Large waterworks | Total |
| --- | --- | --- | --- | --- |
| Waterworks | Total | 2224 | 7223 | 9447 |
| ownership waterworks | vv_municipal | 1886 (85%) | 7096 (98%) | 7096 (95%) |
|  | vv_private | 338 (15%) | 127 (2%) | 127 (5%) |
| Sex | Male | 1068 (48%) | 3386 (47%) | 3386 (47%) |
|  | Female | 1156 (52%) | 3837 (53%) | 3837 (53%) |
| Age | 00-05 | 52 (2%) | 295 (4%) | 295 (4%) |
|  | 06-12 | 79 (4%) | 390 (5%) | 390 (5%) |
|  | 13-18 | 56 (3%) | 192 (3%) | 192 (3%) |
|  | 19-49 | 588 (26%) | 2156 (30%) | 2156 (29%) |
|  | 50-64 | 877 (39%) | 2397 (33%) | 2397 (35%) |
|  | 65-80 | 572 (26%) | 1793 (25%) | 1793 (25%) |
| Education | Tertiary education | 892 (40%) | 3935 (54%) | 3935 (51%) |
|  | Primary, secondary, other education | 1152 (52%) | 2433 (34%) | 2433 (38%) |
|  | Persons below 18 years | 171 (8%) | 833 (12%) | 833 (11%) |
|  | NA | 9 (0,4%) | 22 (0,3%) | 22 (0,3%) |
| Region | South | 151 (7%) | 0 (0%) | 0 (2%) |
|  | East | 452 (20%) | 5322 (74%) | 5322 (61%) |
|  | West | 876 (39%) | 1170 (16%) | 1170 (22%) |
|  | Middle | 232 (10%) | 546 (8%) | 546 (8%) |
|  | North | 513 (23%) | 185 (3%) | 185 (7%) |
| Persons in household | 1 person | 400 (18%) | 1125 (16%) | 1125 (16%) |
|  | 2 persons | 1232 (55%) | 3806 (53%) | 3806 (53%) |
|  | 3 or more persons | 584 (26%) | 2253 (31%) | 2253 (30%) |
|  | NA | 8 (0,4%) | 39 (0,5%) | 39 (0,5%) |
| Children under 7years of age (only 16+) | Yes | 140 (6%) | 679 (9%) | 679 (9%) |
|  | No | 1913 (86%) | 5710 (79%) | 5710 (81%) |
|  | NA | 171 (8%) | 834 (12%) | 834 (11%) |
| Children in daycare (only 16+) | Yes | 147 (7%) | 753 (10%) | 753 (10%) |
|  | No | 2075 (93%) | 6463 (89%) | 6463 (90%) |
|  | NA | 1 (0,04%) | 4 (0,06%) | 4 (0,05%) |
| Other children under 7years of age (only 0-15 years) | Yes | 49 (2%) | 243 (3%) | 243 (3%) |
|  | No | 122 (5%) | 590 (8%) | 590 (8%) |
|  | NA | 2053 (92%) | 6390 (88%) | 6390 (89%) |
| Other children in daycare (only 0-15 years) | Yes | 36 (2%) | 182 (3%) | 182 (2%) |
|  | No | 13 (0,6%) | 61 (0,8%) | 61 (0,8%) |
|  | NA | 2175 (98%) | 6980 (97%) | 6980 (97%) |
| Housing, type household | Farm with production animals | 122 (5%) | 82 (1%) | 82 (2%) |
|  | Single-family-home | 1925 (87%) | 5108 (71%) | 5108 (74%) |
|  | Duplex,Townhouse,Semi-detached house | 98 (4%) | 1318 (18%) | 1318 (15%) |
|  | Apartment building | 18 (0,8%) | 425 (6%) | 425 (5%) |
|  | Other | 49 (2%) | 251 (3%) | 251 (3%) |
|  | NA | 12 (0,5%) | 39 (0,5%) | 39 (0,5%) |
| Wash hands before eating | Always,usually | 1902 (86%) | 5936 (82%) | 5936 (83%) |
|  | Occasionnaly | 224 (10%) | 927 (13%) | 927 (12%) |
|  | Rarely,never | 64 (3%) | 284 (4%) | 284 (4%) |
|  | NA | 34 (2%) | 76 (1%) | 76 (1%) |
| Wash hands before coocking | Always,usually | 2070 (93%) | 6634 (92%) | 6634 (92%) |
|  | Occasionnaly | 64 (3%) | 262 (4%) | 262 (3%) |
|  | Rarely,never | 19 (0,9%) | 72 ( 1%) | 72 ( 1%) |
|  | NA | 71 (3%) | 255 (4%) | 255 (3%) |
| Wash kitchen equipement | Always,usually | 2059 (93%) | 6521 (90%) | 6521 (91%) |
|  | Occasionnaly | 23 (1%) | 109 (2%) | 109 (1%) |
|  | Rarely,never | 57 (3%) | 204 (3%) | 204 (3%) |
|  | NA | 85 (4%) | 389 (5%) | 389 (5%) |
| Farm animals (weekly contact) | Contact with farm animals | 335 (15%) | 470 (7%) | 470 (9%) |
|  | No contact with farm animals | 1889 (85%) | 6753 (93%) | 6753 (91%) |
| Pet/animals (in household) | Pet/animals in household | 1032 (46%) | 2865 (40%) | 2865 (41%) |
|  | No pet/animals in household | 1192 (54%) | 4358 (60%) | 4358 (59%) |
| Contact with wild animals/birds | Contact with wild animals/birds | 987 (44%) | 2295 (32%) | 2295 (35%) |
|  | No contact with wild animals/birds | 1237 (56%) | 4928 (68%) | 4928 (65%) |
| Drinking water from nature | Yes | 1343 (60%) | 3011 (42%) | 3011 (46%) |
|  | No | 841 (38%) | 4067 (56%) | 4067 (52%) |
|  | NA | 40 (2%) | 145 (2%) | 145 (2%) |
